# Epidemiology of HIV, syphilis, and hepatitis B and C among manual cane cutters in low-income regions of Brazil

**DOI:** 10.1186/s12879-018-3439-4

**Published:** 2018-11-03

**Authors:** Déborah Ferreira Noronha de Castro Rocha, Luana Rocha da Cunha Rosa, Carla de Almeida Silva, Brunna Rodrigues de Oliveira, Thaynara Lorrane Silva Martins, Regina Maria Bringel Martins, Marcos André de Matos, Megmar Aparecida dos Santos Carneiro, Juliana Pontes Soares, Ana Cristina de Oliveira e Silva, Márcia Maria de Souza, Robert L. Cook, Karlla Antonieta Amorim Caetano, Sheila Araujo Teles

**Affiliations:** 1 Faculty of Nursing, Federal University of Goias/Universidade Federal de Goiás, Goiânia, GO Brazil; 2 Institute of Tropical Pathology and Public Health, Federal University of Goias/Universidade Federal de Goiás, Goiânia, GO Brazil; 3 Faculty of Nursing, Federal University of Paraiba/Universidade Federal da Paraíba, João Pessoa, PB Brazil; 4 Department of Epidemiology, College of Public Health and Health Professions and College of Medicine, University of Florida, Gainesville, FL USA

**Keywords:** Sexually transmitted diseases, Rural population, Poverty areas, Viral hepatitis vaccines

## Abstract

**Background:**

In recent decades the epidemic of asymptomatic sexually transmitted infections has extended deep into Brazil, including small towns and rural areas. The purpose of this study was to investigate the epidemiology of HIV, syphilis, and hepatitis B (HBV) and hepatitis C viruses (HCV), and to evaluate immunization coverage against hepatitis B in a group of rural workers in Brazil.

**Methods:**

In 2016, a cross-sectional study was conducted with 937 manual sugarcane cutters of the Midwest and Northeast Regions of Brazil. All individuals were interviewed and screened for HIV, syphilis, HBV and HCV. Correlating factors with lifetime HBV infection were investigated using logistic regression. Positive Predictive Values, Negative Predictive Values, sensitivity and specificity were also calculated relative to vaccination against Hepatitis B, comparing anti-HBs titers to vaccination reports.

**Results:**

Most reported previous hospitalization (55%), occupational injuries (54%), sharing of personal items (45.8%), alcohol consumption (77.2%), multiple sexual partners in previous 12 months (39.8%), and no condom use during sexual intercourse in last 12 months (46.5%). Only 0.2% reported using injection drugs. Anti-HIV-1 was detected in three individuals (0.3%). Serological markers of lifetime syphilis (treponemal test) were detected in 2.5% (95% CI: 1.6–3.6) of participants, and active syphilis (treponemal test and VDRL) present in 1.2%. No samples were positive for anti-HCV. The prevalence of lifetime HBV infection (current or past infection) was 15.9%, and 0.7% (95% CI 0.4 to 1.5) were HBsAg-positive. Previous hospitalization (OR 1.53, CI 1.05–2.24, *p* < 0.01) and multiple sexual partners in the last 12 months (OR 1.80, CI 1.25–2.60, *p* < 0.01) were predictors for lifetime HBV infection. Although 46.7% (95% CI 43.4–49.9) of individuals reported having been vaccinated against hepatitis B, only 20.6% (95% CI 18.1–23.3) showed serological evidence of previous hepatitis B vaccination (positive for anti-HBs alone).

**Conclusions:**

The high prevalence of syphilis and HBV compared to the general population and the high frequency of risk behaviors show the potential for sexual and parenteral dissemination of these agents in this rural population. In addition, the low frequency of hepatitis B vaccinated individuals suggests a need for improved vaccination services.

## Background

Despite advancements in prevention and diagnosis, asymptomatic sexually transmitted infections are a major challenge for global infection control [1, 2]. Worldwide, 36.7 million people are living with HIV [3], while approximately 5.6 million new cases of syphilis occur each year [4]. Hepatitis B and C, in turn, are also a major public health challenge, and 325 million people are chronic carriers of these viruses [2]. Although globally the hepatitis B vaccine has decreased the burden of hepatitis B virus infection significantly, most adult individuals remain susceptible to HBV [2].

The distribution of asymptomatic sexually transmitted infections varies worldwide, and studies have shown that social, economic and behavioral conditions influence their epidemiology [4, 5]. Therefore, in general, higher prevalences of HIV, syphilis, and hepatitis B and C have been found in low- and middle-income countries compared to developed countries [4, 6].

In Brazil, the epidemic of HIV infection started in the industrialized regions of the southeast and south, and vulnerable urban groups have the highest burden of this infection [7–9]. However, in the last decade the HIV epidemic has reached inner cities and rural regions, far from the epicenter of the epidemic, where access to health services is limited and often low quality [10]. Further, HIV infection is often accompanied by other asymptomatic sexually transmitted infections such as syphilis and viral hepatitis, which, like HIV, have great potential for dissemination in impoverished vulnerable populations [1]. There are few studies in Brazil on the epidemiology of these infections in less populated regions of the country [8, 10].

Brazil is the largest producer of sugar cane in the world, and sugar cane-based industries are a significant economic activity. Brazil’s sugar-alcohol industry employs around 500,000 workers linked exclusively to sugarcane [11] and almost 90% of production takes place in the Central and Southern regions of the country, followed by the Northeast region [12]. Although the mechanization of sugarcane cutting has been expanded in the last decade, this trend occurred predominantly in the Southern Region. In the Midwestern and Northeastern region, manual cane cutting remains the principal mode of cane harvesting. This activity is physically and mentally taxing, and despite advances in working conditions, these individuals still experience exploitation and dangerous working conditions as well as social marginalization [13].

Due the nature of sugar cane cutting, these workers are usually males, young adults, and sexually active [14]. The seasonality of sugarcane cultivation and the possibility of better income encourages the temporary inter-regional migration of workers, who live their lives isolated from their families and partners for months. Most of them live in large communal houses on sugarcane farms, where the sharing of personal care items is frequent. Further, on their days off they visit the cities or villages nearby sugarcane plantations to buy personal items and have fun, which can include binge drinking and unsafe sexual encounters [15].

This corollary of factors may put manual sugar cane cutters at high risk of asymptomatic sexually transmitted infections, and they could be potential disseminators of these infections in remote regions. However, data about the epidemiology of these vulnerable rural workers is virtually non-existent in Brazil. This study estimated HIV, viral hepatitis B and C, and syphilis prevalence, and analyzed risk factors for lifetime HBV infection, and evaluated immunization against hepatitis B in sugarcane cutters in Central and Northeastern Brazil.

## Methods

This is a cross-sectional, multicenter study in Goiás (Midwest Region) and Paraíba (Northeast Region), Brazil. Goiás has an estimated population of 6,778,772, monthly nominal household income per capita of $348 and a Human Development Index (HDI) of 0.735. Paraíba has an estimated population of 4,025,558 inhabitants, monthly nominal household income per capita of $240 and HDI of 0.658. The agricultural industry that has been developed in both states has great significance to the national economic situation [16].

In Goiás there are 38 alcohol and sugar producing units distributed across different regions of the state, and in 14 of them manual cane harvesting is still used. Among them, four were in harvest activity during the period of the study, and they were included in the investigation. In Paraíba there are nine alcohol and sugar production plants, only one sugar producing unit was eligible, being that It used manual cutting and was active [17]. This is the largest sugar producing unit in the State.

The minimum required sample, considering a statistical power of 80% (β = 20%), significance level of 95%, (< 0,05), precision of 0.5%, design effect of 1.5 and a global prevalence for anti-HIV for the general population at 0.39% [18] was 895 manual sugar cane cutters.

**Fig. 1:**
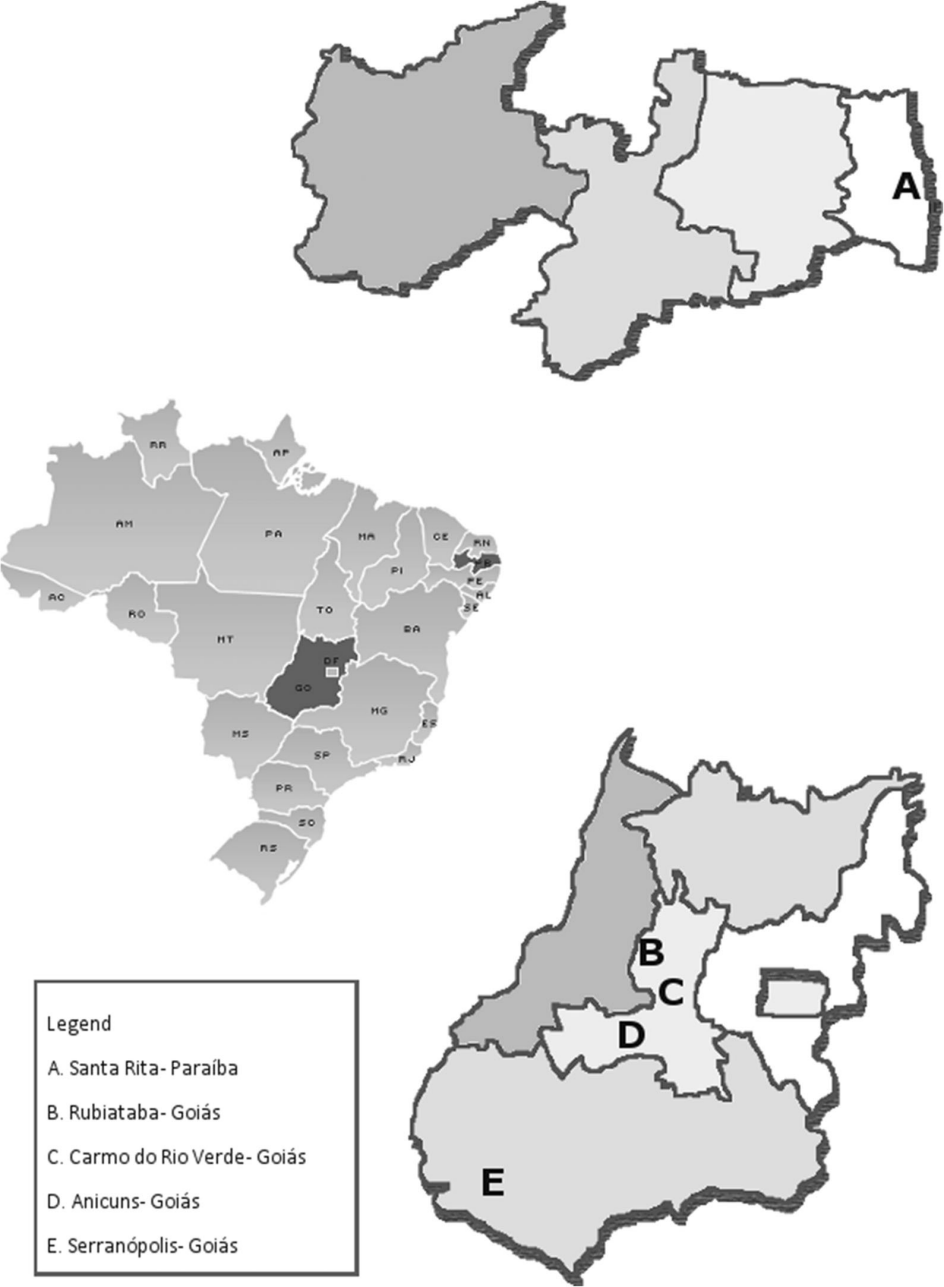
Geographical location of the sampling sites in the states of Goiás (GO) and Paraíba (PB), Central-West and Northeast regions, respectively

Inclusion criteria were: be a manual sugar cane cutter (by self-report) and be aged 18 years or older. Five alcohol and sugar producing units agreed to participate in the study, representing the manual sugar cane cutters from these regions (Fig. 1).

Data collection was carried out from February to September 2016. All participants signed a free and informed consent form, approved by the research ethics committee of the Federal University of Goiás and Paraíba, according to resolution CNS No. 466/12, under protocols 042796/2015 and 1,507,737/2016, respectively. Participants were interviewed by trained research assistants using a structured script containing questions about sociodemographic characteristics, work characteristics, risk behaviors for sexually transmitted infections (STIs), also referred to as sexually transmitted diseases (STDs) and viral hepatitis, as well as vaccination status. “Reported Shared Accommodation” was defined as individuals who shared their living space with roommates or flatmates, or in shared employer-provided housing.

Rapid screening tests for HIV (Bioeasy HIV, Republic of Korea) and syphilis (Alere Syphilis, Republic of Korea) were performed on all participants, as well as confirmatory rapid tests for HIV (Abon™ HIV 1/2/O, China). For complementary serological tests for syphilis, hepatitis B, and C, 10 ml of blood were collected.

For syphilis, positive samples per the treponemal test (Rapid Test) were retested by a non-treponemal test - venereal disease research laboratory (VDRL) (Wiener Lab, Argentina). For study purposes, active syphilis was defined as the participant being positive for both tests [19, 20]. For hepatitis B and C, all samples were tested for HBsAg, anti-HBs, total anti-HBc, and anti-HCV by enzymatic immunoassay using Biokit S.A., Spain. Persons with positive tests were referred for diagnostic confirmation and/or treatment.

Interview data and serological test results were digitized and analyzed using the STATA statistical software version 13.0 (StataCorp, College Station, TX). Prevalences were calculated with 95% confidence intervals. Univariate and multivariate analyses were performed to identify factors associated with lifetime HBV infection. The term “lifetime HBV infection” refers to the presence of the HBsAg and/or anti-HBc markers, indicating current or past HBV infection. Therefore, for the purposes of analysis, cases that showed isolated positivity for anti-HBs were excluded. Initially, risk factors for seropositivity to lifetime HBV infection were estimated. Those factors that presented a statistically significant association (*p* < 0.20) were subjected to multivariate Logistic regression analysis and controlled by “work region”. The Chi-squared, Chi-squared test for trend, Fisher’s exact test, and student’s t tests were used to evaluate differences between proportions. The significance level used in the tests was 5%. Positive Predictive Values (PPV) and Negative Predictive Values (NPV) were also calculated for sensitivity and specificity related to self-reported vaccination against Hepatitis B (complete series), considering the serological profile of immunization as the gold standard (i.e. HBsAb positive only).

## Results

A total of 937 manual sugar cane cutters were recruited and agreed to participate in the study (Table 1). All were male and 68.9% were under 40 years of age. Regarding education, almost half (47.4%) reported having 4 or fewer years of education. Most participants were married (77.5%). In terms of region, 85.7% were from the Northeast of the country; however, 67.9% worked in plants located in the Central region of Brazil. The average monthly salary was $554.10.

**Table 1:** Sociodemographic characteristics of manual sugar cane cutters in the Northeast and Central regions of Brazil, 2016 (*n* = 937)

Variables	Participants	%
	*n* = 937	
Sex		
Male	937	100.0
Age^a^ mean (standard deviation)	35.4 (9.2)	
= < 29	266	28.4
30–39	380	40.5
= > 40	291	31.1
Education^a^ mean (standard deviation)	5.2 (3.6)	
< = 4	444	47.4
> 4	493	52.6
Civil Status		
Married	726	77.5
Single	211	22.5
Region of Origin		
Northeast	803	85.7
Midwest	129	13.8
North	4	0.4
Southeast	1	0.1
Work region		
Paraíba	301	32.1
Goiás	636	67.9
Income^b^ mean (standard deviation)	554.1 (165.7)	
≤ 461	326	34.8
462–615	412	44
> 616	199	21.2

^a^Years; ^b^US$/per month

Table 2 shows risk factors for sexual and bloodborne pathogens reported by the sugar cane cutters in this study (Table 2). Most reported previous hospitalization (55%), occupational injuries (54%), sharing of personal items – such as razor blades, nail pliers, toothbrushes (45.8%), alcohol consumption (77.2%), age of first sexual intercourse before 16 years old (49.4%), multiple sexual partners (last 12 months) (39.8%), and no condom use during sexual intercourse (last 12 months) (46.5%).

**Table 2:** Risk factors for sexual and bloodborne pathogens in manual sugar cane cutters in the Northeast and Central regions of Brazil, 2016 (*n* = 937)

Variables	*N*	%
Knowledge of signs or symptoms of STI in women (no)	639	68.2
Knowledge of signs or symptoms of STI in men (no)	600	64
Tattoos or piercings on body (yes)	106	11.3
Previous transfusion (yes)	38	4.1
Previous hospitalization (yes)	515	55
Previous occupational injuries (yes)	505	54
Reported shared accommodation (yes)	277	29.6
Sharing sharp personal care items (yes)	429	45.8
History of incarceration (yes)	90	9.6
History of drug use (yes)	126	13.4
History of marijuana use (yes)	110	11.7
History of cocaine/crack use (yes)	43	4.6
History of injection drug use (yes)	2	0.2
Drinking alcohol (yes)	723	77.2
Age of first sexual intercourse = < 15 years (15.7; 2.7)^a^	463	49.4
History of STI (yes)	101	10.8
History of homosexual relations (yes)	52	5.5
History of sexual abuse (yes)	13	1.4
Sexual partner number = > 2 in the last 12 months (2.8; 4.6)^a^	373	39.8
No condom use at least once in the past 12 months	436	46.5
Report of genital ulcer/sore in the last 12 months (yes)	34	3.6
Report of genital discharge in the last 12 months (yes)	29	3.1

^a^Mean; standard deviation

Anti-HIV-1 was detected in three individuals (0.3%; 95% CI: 0.1–1.0). Serological markers of lifetime syphilis (Treponemal test) were detected in 2.5% (95% CI: 1.6–3.6) of sugar cane cutters, and of active syphilis (Treponemal test and VDRL) in 1.2% (95% CI: 0.6–2.1). The prevalence of lifetime HBV infection was 15.9% (95% CI: 13.7–18.4), where 0.7% (95% CI: 0.4–1.5) were HBsAg-positive, 9.8% (95% CI: 8.1–11.9) were for both anti-HBs and anti-HBc markers, and 5.3% (95% CI: 4.1–7.0) were positive for anti-HBc alone. No samples tested positive for anti-HCV (Table 3).

**Table 3:** Prevalences of HBV, HCV, HIV, and Syphilis serological markers in manual sugar cane cutters in the Northeast and Central regions of Brazil, 2016 (n = 937)

Serological markers	Positive		(CI 95%)^a^
	*n*	%	
Isolated HBsAg	1	0.1	0.0–0.6
HBsAg + anti-HBc	6	0.6	0.3–1.4
Anti-HBs + anti-HBc	92	9.8	8.1–11.9
Anti-HBc alone	50	5.3	4.1–7.0
Lifetime HBV infection	149	15.9	13.7–18.4
Anti-HBs alone	193	20.6	18.1–23.3
Anti-HCV	0	–	
Lifetime Syphilis (TT)^b^	23	2.5	1.6–3.6
Active Syphilis (TT^b^ and VDRL^c^)	11	1.2	0.6–2.1
Anti-HIV	3	0.3	0.1–1.0

^a^CI: Confidence Interval ^b^TT: treponemal test ^c^*VDRL* Venereal disease research laboratory

Among those sugar cane cutters positive for HBsAg and/or anti-HBc (*n* = 149), 4% (6/149) of the subjects were also positive by the Treponemal tests, 2% (3/149) for VDRL and one for anti-HIV-1 (0.7%).

In univariate analyses for lifetime HBV infection, six variables showed *p* value < 0.20 and were included in the multivariate model: civil status, history of incarceration, history of marijuana use, history of cocaine/crack, previous hospitalization, age at first sexual intercourse, history of STI, number of sexual partners and no condom use at least once in the past 12 months. The final model showed previous hospitalization (OR 1.53, CI 1.05–2.24, *p* = 0.027) and multiple sexual partners in the last 12 months (OR 1.8, CI 1.25–2.60, *p* < 0.01) were predictors for lifetime HBV infection among the sugar cane cutters investigated (Table 4).

**Table 4:** Univariate and multivariate analyses of risk factors associated with HBV of sugar cane cutters, Northeast and Central regions of Brazil, 2016

Variable	Univariate analysis^a^				Multivariate analysis^a^	
	HBV		Pvalue	OR^b^(95% CI)	HBV	
	Positive	Negative			Pvalue	OR^b^ (95% CI)
	(n = 149)	(595)				
Age (years) (36.7; 8.9)^c^						
18–29	31 (19.4%)	129 (80.6%)		1		
30–39	65 (20.4%)	254 (79.6%)	0.796	1.06 (0.66–1.71)		
> =40	53 (20%)	212 (80%)	0.875	1.04 (0.63–1.70)		
Education (years) (4.7; 3.5)^c^						
< =4	74 (19.2%)	311 (80.8%)		1		
> 4	75 (20.9%)	284 (79.1%)	0.569	1.10 (0.77–1.59)		
Civil Status						
Married	110 (18.5%)	484 (81.5%)		1		1
Single	39 (26%)	111 (74%)	0.042	1.54 (1.02–2.35)	0.300	1.27 (0.81–1.98)
Reported shared accommodation						
No	112 (21%)	422 (79%)		1		
Yes	37 (17.6%)	173 (82.4%)	0.304	0.80 (0.53–1.22)		
Work region						
Northeast	44 (17.7%)	204 (82.3%)		1		
Midwest	105 (21.2%)	391 (78.8%)	0.271	1.24 (0.84–1.84)		
History of incarceration						
No	129 (19.3%)	541 (80.7%)		1		1
Yes	20 (27%)	54 (73%)	0.115	1.55 (0.89–2.69)	0.122	1.55 (0.89–2.70)
History of marijuana use						
No	136 (20.9%)	516 (79.1%)		1		1
Yes	13 (14.1%)	79 (85.9%)	0.134	0.62 (0.34–1.15)	0.084	0.58 (0.31–1.08)
History of cocaine/crack use						
No	147 (20.7%	564 (79.3%)		1		1
Yes	2 (6.1%)	31 (93.9%)	0.058	0.25 (0..6–1.05)	0.067	0.26 (0.06–1.10)
Tattoos or piercings on body						
No	131 (19.8%)	530 (80.2%)		1		
Yes	18 (21.7%)	65 (78.3%)	0.689	1.12 (0.64–1.95)		
Drinking alcohol						
No	29 (16.8%)	144 (83.2%)		1		
Yes	120 (21%)	451 (79%)	0.222	1.32 (0.84–2.06)		
Previous transfusion						
No	144 (20.1%)	574 (79.9%)		1		
Yes	5 (19.2%)	21 (80.8%)	0.918	0.95 (0.35–2.56)		
Previous hospitalization						
No	54 (16.2%)	280 (83.8%)		1		1
Yes	95 (23.2%)	315 (76.8%)	0.018	1.56 (1.07–2.26)	0.027	1.53 (1.05–2.24)
Previous work accident						
No	64 (18.5%)	281 (81.5%)		1		
Yes	85 (21.3%)	314 (78.7%)	0.350	1.18 (0.83–1.71)		
Sharing sharp personal care items						
No	78 (18.8%)	337 (81.2%)		1		
Yes	71 (21.6%)	258 (78.4%)	0.346	1.18 (0.83–1.70)		
Age at first sexual intercourse (years) (15.7; 2.7)^c^						
7–15	86 (22.9%)	289 (77.1%)		1		1
> =16	63 (17.1%)	306 (82.9%)	0.046	0.69 (0.48–0.99)	0.116	0.74 (0.51–1.07)
History of STI						
No	126 (18.9%)	539 (81.1%)		1		1
Yes	23 (29.1%)	56 (70.9%)	0.035	1.75 (1.04–2.96)	0.088	1.59 (0.93–2.72)
History of homosexual relations						
No	139 (19.8%)	564 (80.2%)		1		
Yes	10 (24.4%)	31 (75.6%)	0.474	1.30 (0.62–2.73)		
History of sexual abuse						
No	146 (19.9%)	589 (80.1%)		1		
Yes	3 (33.3%)	6 (66.7%)	0.325	2.02 (0.49–8.16)		
Number of sexual partners in the last 12 months (2.6; 3.4)^c^						
< =1	74 (16.2%)	383 (83.8%)		1		1
> =2	75 (26.1%)	212 (73.9%)	0.001	1.83 (1.27–2.63)	0.002	1.8 (1.25–2.60)
No condom use at least once in the past 12 months						
No	84 (22.2%)	295 (77.8%)		1		1
Yes	65 (17.8%)	300 (82.2%)	0.138	0.76 (0.53–1.09)	0.866	1.04 (0.67–1.61)
Report of genital ulcer/sore in the last 12 months						
No	140 (19.6%)	573 (80.4%)		1		
Yes	9 (29%)	22 (71%)	0.205	1.67 (0.75–3.72)		
Report of genital discharge in the last 12 months						
No	144 (20.1%)	574 (79.9%)		1		
Yes	5 (19.2%)	21 (80.8%)	0.918	0.95 (0.35–2.56)		

^a^Logistic Regression, adjusted by work region; ^b^Odds Ratio; ^c^Mean; standard deviation

Although 46.7% (95% CI: 43.4–49.9) of individuals reported having been vaccinated against hepatitis B, only 20.6% (95% CI: 18.1–23.3) showed serological profile of previous hepatitis B vaccination (positive for anti-HBs alone) and the mean age was 30.7 years (SD: 8.5). Therefore, self-reported previous HBV vaccination reports showed a positive predictive value and specificity to identify individuals immunized against hepatitis B of only 27.6% and 57.4%, respectively. The negative predictive value and sensitivity of the vaccination report were 85.6% and 62.7%, respectively.

## Discussion

In Brazil, the epidemic of HIV/AIDS is moving from urban centers and reaching small cities and villages [10], and this investigation supports this dynamic. Most rural workers were poor seasonal migrants with low education, from small cities of the poorest regions of Brazil (North and Northeast). In fact, the anti-HIV-1 prevalence found among these sugar cane cutters was similar to that found in the general population in Brazil and worldwide. In 2016, the World Health Organization reported a prevalence of 0.8% [0.7–0.9%] in adults [21], and in Brazil, a rate of 0.39% among people aged 15 to 49 years is estimated [18].

The potential of HIV dissemination in the study population may be measured by the prevalence of other sexually transmitted infections. Unlike HIV, the lifetime syphilis prevalence found among study participants was higher than that estimated in the general population worldwide and in Brazil [18, 22]. A meta-analysis including data of 154 countries showed a global pooled mean prevalence of 1.11% (95% CI: 0.99–1.22). Further, when only studies carried out in American regions were considered, the prevalence decreased to 0.13 (95% CI: 0.09–0.19) [20]. In Brazil, a survey carried out among 35,460 Brazilian male conscripts found a lifetime syphilis prevalence of 0.55% (95% CI: 0.45–0.61) [23].

Concerning hepatitis B, though the prevalence of lifetime HBV infection suggests a low HBV endemicity among the sugarcane cutters investigated, it should be emphasized this prevalence was slightly higher than that estimated in the urban population (20 to 69 year) from the Midwest (12.4%; 95% CI: 11.1–14.3) and Northeast (12.1%; 95% CI: 10.5–13.9) regions of Brazil [24]. On the other hand, this prevalence was similar to that reported recently in populations at risk for STIs, such as sex workers (17.1%; 95% CI: 11.6–23.4) [25] and men who have sex with men (MSM) (15.4%; 95% CI: 8.7–25.8) in Goiás [26]. In addition, seven individuals were HBsAg positive, being therefore potential disseminators of HBV. Further, six individuals had been infected by HBV and *T. pallidum*, and three of them had active syphilis. One sugar cane cutter had been infected by HBV and HIV.

The asymptomatic characteristics of these infections favor their quiet dissemination. In the absence of knowledge of these diseases, diagnosis and treatment, they scatter efficiently in vulnerable populations that present risk behaviors, like manual sugarcane cutters [27, 28]. In this study, these conditions favorable to STI dissemination were present. The average education level reported was only 4 years of study and few had knowledge about STIs and access to public health services. Indeed, of the total, 40% had not sought health services in the last 12 months, and 30% had only sought health services one time (data not shown).

The analyses of potential risk factors for HBV identified two predictors: multiple partners and previous hospitalization. HBV sexual transmission is well established [2], and supports the potential of sugar cane cutters as disseminators of other STIs, including HIV. In fact, the use of condoms during sexual intercourse is not a regular practice and the consumption of alcohol is high in this population. These behaviors have encouraged the spread of STIs [1, 29].

In this investigation, the high frequency of previous hospitalization (*n* = 520) was a surprise, and we could speculate that this occurred due to workplace hazards including those that present multiple health-risk situations [30]. In this study, 509/943 individuals reported an occupational injury, and 156/943 suffered one in the last 12 months. These rural workers are exposed to long daily shifts and numerous injuries, including stress, dehydration, bites of venomous animals, accidents, burns by sunburn or by fire, poisoning by pesticide residues, etc. [30–32]. Sometimes these situations require health care and hospitalizations, which in low-income regions may be a cause of HBV infection [33]. This is often a consequence of a lack of resources to perform proper hygiene as well as staff trained in proper patient safety procedures. Brazil is a continental country with large economic and cultural diversity [34]. Therefore, the findings of previous hospitalization as a predictor of lifetime HBV infection among sugar cane cutters, suggest HBV dissemination in healthcare facilities where infection control measures may be a luxury where qualified human resources, equipment, and supplies are scarce, favoring parenteral transmission. In fact, previous studies conducted in Brazil have also shown an association between invasive medical procedures and HBV infection [35–37].

Hepatitis B vaccine is the main preventive measure against HBV infection [2]. In Brazil, currently, the HBV vaccine is available free of charge for the entire population [22]. Despite this policy, vaccine coverage is still low among adults, mainly men [38]. In this investigation, only 20.6% of sugarcane cutters had isolated anti-HBs protective titers, indicating previous immunization. Low education, low-income and lack of public health services very probably contributed to these findings. In fact, some authors have shown that individuals with a greater understanding of the disease and with a higher economic level tend to be vaccinated against hepatitis B compared to those who are unaware of hepatitis B vaccine [39].

The best information about an individual’s previous vaccination is their vaccination card [40]. If it is not available, many health professionals trust in the verbal report of previous vaccination [41]. However, this study supports that a hepatitis B vaccination self-report is not an accurate indicator of previous vaccination status. In fact, our findings showed that a self-report of HBV vaccination had a sensitivity and specificity of 62.7% and 57.4% to identify individuals immunized against HBV, respectively. In a study conducted in the United States with 818 individuals of an integrated health care system, better results were found (sensitivity and specificity of 73% and 67%, respectively), but the social differences between these two populations are noteworthy, including education and income [42]. Therefore, a report of previous hepatitis B vaccination should not be considered an indicator of hepatitis B immunization, mainly in impoverished populations. For these populations, in the absence of the vaccination card, “Don’t Ask, Vaccinate”! [43].

There were no cases of hepatitis C among the individuals investigated. HCV is predominantly transmitted by blood. Therefore, transfusion of unscreened blood and sharing of syringes and needles among drug addicts has been the major cause of HCV transmission [2]. However, these were uncommon among the individuals studied and should explain in part our findings.

This investigation has some limitations. Initially, due to financial restrictions the data collection was carried out in February and March in Paraíba, Northeast Region, which is the period at the end of the sugar cane harvest season. Therefore, only one mill was included in the study. However, this represents the biggest mill in the region. Further, the study was conducted in only two states, and may not represent all Brazilian manual sugar cane cutters, although these two states represent the most significant alcohol producing areas in Brazil. All interviews were performed face-to-face, therefore some personal and private questions may have biased responses, and therefore underestimate the prevalence of such sensitive variables as condom use, sex with men who have sex with men (MSM), illicit drug use, etc. Otherwise, some strategies were used to minimize potential biases: previously trained male interviewers, and private places for interviews.

## Conclusion

This research presents the situation of poor, rural workers from non-industrialized areas of Brazil, a population often disregarded from a public health standpoint. The situation of these young men includes wide circulation of HIV and high prevalences of syphilis and HBV compared to the national population. In addition, the variables associated with HBV infection, multiple partners and previous hospitalization, showed the risk of dissemination of sexually and parenterally transmitted infections in this rural population. Finally, the low frequency of individuals vaccinated against hepatitis B suggests a need for improved vaccination services. It is therefore recommended that sugar and ethanol plants act to strengthen specific prevention and health promotion programs for rural sugarcane workers in Brazil.

## Abbreviation

CI: Confidence Interval
HBV: Hepatitis B virus
HCV: Hepatitis C virus
HDI: Human development index
HIV: Human immunodeficiency virus
MSM: Men who have sex with men
NPV: Negative predictive values
PPV: Positive predictive values
STD: Sexually transmitted disease
STI: Sexually transmitted infection
TT: Treponemal test
VDRL: Venereal disease research laboratory
